# Early warning predictions of contaminants in animal feed using machine learning

**DOI:** 10.1038/s41538-025-00634-1

**Published:** 2025-11-21

**Authors:** L. M. van den Bulk, R. G. Hobé, W. Hoenderdaal, D. Giržadas, A. Nijrolder, H. J. van der Fels-Klerx

**Affiliations:** Wageningen Food Safety Research, Wageningen, The Netherlands

**Keywords:** Environmental sciences, Risk factors

## Abstract

Contaminated feed can lead to negative health effects in both animals and humans, as well as result in economic losses. In order to support risk-based monitoring of animal feed, it is essential that the potential presence of food safety hazards can be predicted in an accurate and timely manner. This study used machine learning to predict the presence of four contaminant groups in animal feed: mycotoxins, heavy metals, dioxins and pesticides. Emphasis was placed on a holistic design, using a broad range of external drivers that may affect the presence of the contaminants as model input. Data included historical feed safety monitoring data related to a wide range of different animal feeds, countries, and years (2010–2023) as well as a variety of socioeconomic and weather indicators related to the country of origin. The CatBoost algorithm was the best performing machine learning model and was used to predict the probability that a contaminant in a feed product from a specific country exceeds a predefined threshold (based on European legal limits or guidance values). The average sensitivity was 83% and average specificity was 73% across the four contaminant groups on a year not seen during model training. The results of the model predictions and data descriptions of the monitoring data were incorporated in a decision support system, which has been tested for use in practice. This system offers risk managers better insights in the potential presence of chemical contaminants in animal feed and supports them in the decision-making to safeguard food safety.

## Introduction

The production of feed, encompassing multiple stages and actors, is vulnerable to contamination with food safety hazards, including microbiological, chemical and/or physical hazards^1^. Unsafe feed may lead to potential negative health effects for both animals and humans, and related economic losses^2^. Feed safety affects human health, as a large portion of human food is derived from animal products, and hazards present in the feed may be carried over to animal products such as meat, milk, or eggs. For instance, aflatoxin B1 present in maize used in dairy cow’s compound feed is transferred into the cow’s milk as aflatoxin M1 with a transfer rate of between 2–6%^3^. To protect human health, threshold levels have been set for the maximal concentration of a variety of safety hazards, mainly chemical contaminants, in food and feed at the European level with legal maximum limits and guidance levels^4–6^.

In addition to these public standards, private standards for feed safety and quality have been defined and are largely applied in feed industry. For example, the Global Partnership for Good Agricultural Practices (GlobalGAP), SecureFeed standards, and Good Manufacturing Practices (GMP) with the integration of Hazard Analysis Critical Control Points (HACCP) have been implemented for primary production and consecutive stages of feed production^7^. Furthermore, monitoring and testing of feed samples for the presence of food safety hazards is applied by industry as part of HACCP procedures, as well as by governmental agencies for official control^8^. However, despite management measures in place to ensure safety in the feed production chain, the presence of hazards may still occur, unexpectedly, due to external drivers such as weather or trade disruptions that may either directly or indirectly affect the presence of safety hazards in animal feed.

Being able to predict the potential presence of safety hazards in animal feed at an early stage would allow more time to risk managers to anticipate and apply mitigation measures to safeguard food safety. As part of this, risk-based monitoring can be applied, allowing available resources to be allocated in an efficient way. In previous studies, different methodologies have been applied to estimate the presence of chemical hazards in animal feed. Van der Fels-Klerx et al.^9^ developed a semi-quantitative model to identify feed ingredients at highest risk to animal and human health for particular groups of contaminants. The model is based on usage and contamination probability of ingredients in conjunction with their health impact factors. It has been applied to dioxins^9^ and the mycotoxins deoxynivalenol and aflatoxin B1^10^. For mycotoxins in grains specifically, predictive models have been developed to estimate the mycotoxin contamination during grain harvest using mainly weather variables as input. Different types of models have been developed, including mechanistic models^11–13^, empirical models^14,15^ and machine learning models^16–19^.

The use of machine learning techniques for the prediction and monitoring of food safety hazards has been growing rapidly, since it can incorporate various data sources, and deal with knowledge gaps and uncertainties. The predictive accuracy can be very high, showing that machine learning models are a promising method to predict safety hazards^20^. With the use of machine learning models, factors from outside the production system, that (in)directly may affect the presence of safety hazards, could be considered as well, in addition to the factors from inside the supply chain. This so-called holistic approach recently has been applied by Liu et al.^21^, where a combination of factors from outside and within the supply chain were used to predict the presence of food safety hazards in milk. In addition, Decision Support Systems (DSS) for the management of food safety hazards are starting to emerge. These systems are designed to help users make better decisions on hazard prevention and control. For example, a DSS for integrated management of mycotoxins in feed and food supply chains was recently developed^22^.

A machine learning model to predict the presence of various chemical safety hazards in a wide variety of animal feed does currently not exist. Therefore, the objective of this study was to develop predictive models for four different contaminant groups (mycotoxins, heavy metals, dioxins and pesticides) in animal feed using a holistic approach, and to integrate the models into a user-friendly DSS to assist experts in their decision-making.

## Results

### Data analysis

**Fig. 1:**
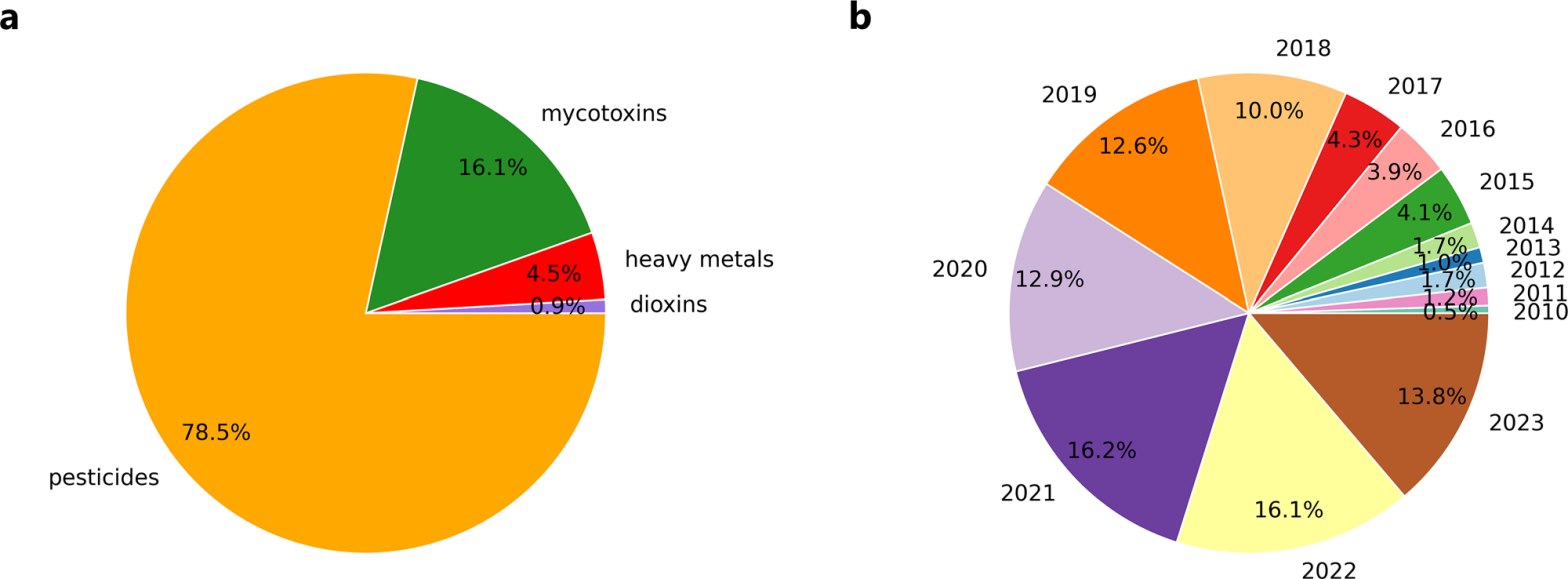
Data analysis of the records in terms of contaminant group and year. **a** The percentage of records per contaminant group (mycotoxins, heavy metals, dioxins, pesticides). **b** The percentage of records per year (2010–2023).

Historical feed monitoring data was combined with open source socioeconomic and weather data to create input for the machine learning models. The created dataset included analytical results for 202,325 different feed samples. Most samples were analyzed for multiple hazards, the dataset consisted of 284,575 mycotoxins records, 79,820 heavy metals records, 15,965 dioxins records, and 1,385,588 pesticides records. Figure 1 presents the percentage of records per contaminant group (panel a) and the percentage of records over sampling years (panel b) of the dataset. Pesticides formed the majority of the records (78.5%), followed by mycotoxins (16.1%), heavy metals (4.5%) and dioxins (0.9%). From Fig. 1b it can be seen that the number of records increased over the analyzed years.

The dataset consisted of a total of 614 different feed products. The top five feed products that occurred most across the samples in the dataset were: maize (37.2%), wheat (9.1%), barley (5.8%), complementary milk cattle feed (4.4%) and maize gluten feed (2.3%). These five products accounted for over half of the total number of samples (58.8%). Furthermore, the combination of specific contaminants and feed products that occurred most across the records were the mycotoxins aflatoxin B1 (3.3%), deoxynivalenol (1.1%), zearalenone (1.0%), and ochratoxin A (0.7%) in maize, in addition to deoxynivalenol (0.8%) and zearalenone (0.7%) in wheat.

**Fig. 2:**
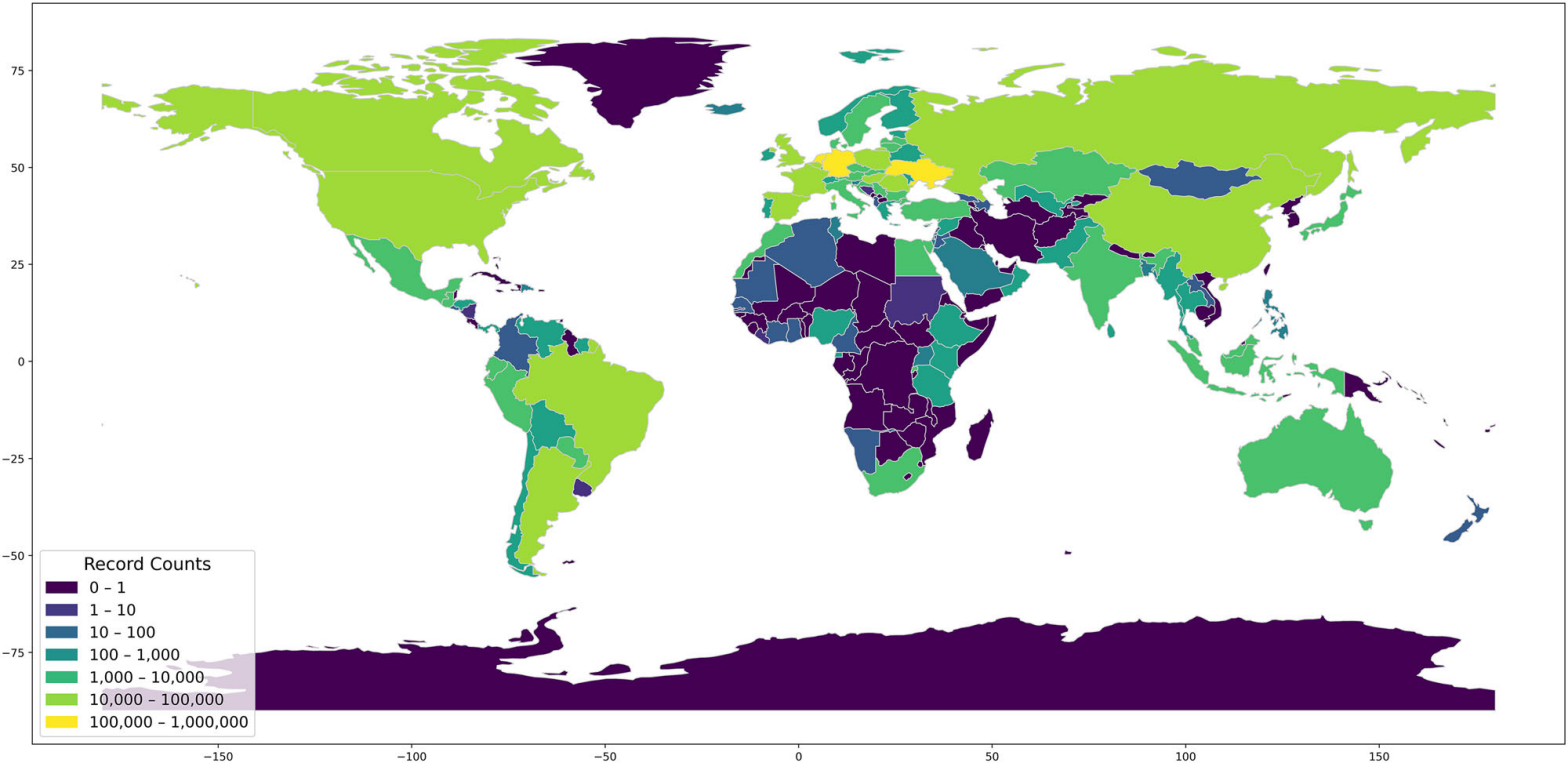
Records per country. The number of records per country of origin on a logarithmic scale.

Figure 2 presents the number of records from the different countries of origin, visualized on a logarithmic scale. Most of the records originated from the Netherlands, Germany, Ukraine, Brazil and Poland. Note that for a bit more than one third of the records (34.7%) the country of origin was not known.

### Contaminant thresholds

A lower threshold than the legal limits or guidance values was chosen to indicate contaminant presence when developing the machine learning models, due to the very limited amount of exceedances. For mycotoxins, heavy metals and dioxins, half of the respective legal limit or guidance levels of the contaminants was considered as the predefined threshold. For pesticides, the records were aggregated by combining the analytical results for the different pesticide residues per sample. This resulted in a total of 17,314 aggregated pesticide records.

Table 1 presents the percentage of records above the predefined exceedance thresholds per contaminant group in the dataset, together with the contaminants present in each group and the percentage of records above the legal limit or guidance value. The percentage of records exceeding this threshold was 2.8% for mycotoxins, 2.2% for heavy metals, and 4.2% for dioxins. For pesticides, for which the MRL per pesticide was used, 6.1% of the aggregated records exceeded the threshold for at least one of the pesticides.

**Table 1:** The percentage of records above the predefined exceedance threshold for each of the contaminant groups

Contaminant group	Contaminants	Exceedance threshold	Percentage above exceedance threshold	Percentage above legal limit/guidance value
Mycotoxins	Aflatoxin B1, Deoxynivalenol, Fumonisin B1 + B2, Ochratoxin A, T2 + HT2 toxin, Zearalenone	Half of the maximum level or guidance value	2.8%	1.4%
Heavy metals	Arsenic, Cadmium, Lead, Mercury	Half of the maximum level	2.2%	0.4%
Dioxins	Toxic Equivalency (TEQ) based on WHO-2005 Toxic Equivalency factors (TEFs) of 17 dioxin congeners (polychlorinated biphenyls were not included)	Half of the maximum level	4.2%	0.6%
Pesticides (Aggregated)	Measurements of pesticide residues (on average 78) were aggregated per sample	MRLs	6.1%	6.1%

### Machine learning models

**Fig. 3:**
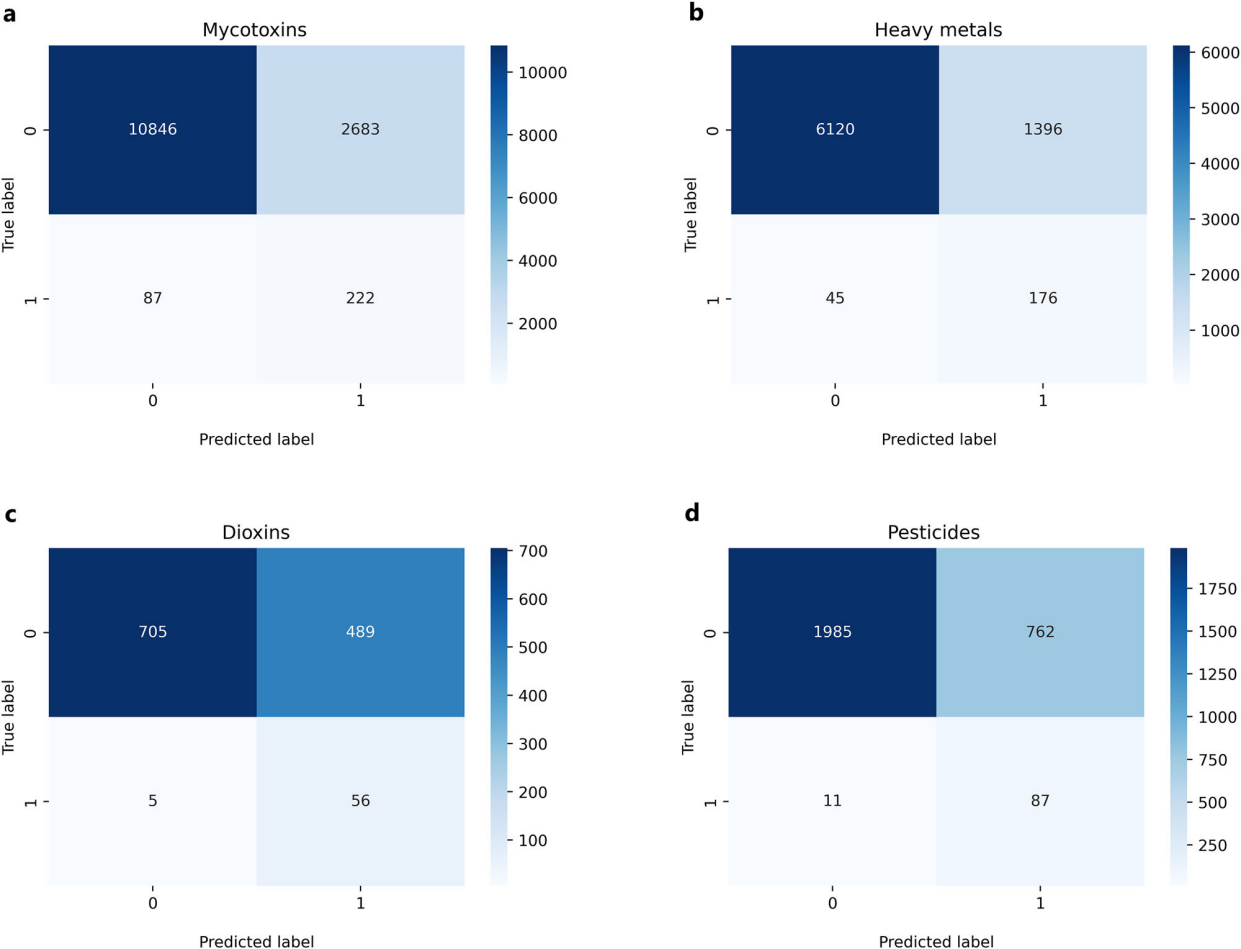
Confusion matrices showing the test set results (2023). **a** The confusion matrix of the test set results from the mycotoxins model. **b** The confusion matrix of the test set results from the heavy metals model. **c** The confusion matrix of the test set results from the dioxins model. **d** The confusion matrix of the test set results from the pesticides model.

Separate CatBoost machine learning models were trained for mycotoxins, heavy metals, dioxins and (aggregated) pesticides to predict whether a contaminant would exceed the predefined exceedance threshold. Results on the test set (2023) of the best-performing models, selected based on their performance on the validation set, can be found in the confusion matrices in Fig. 3 for mycotoxins (panel a), heavy metals (panel b), dioxins (panel c) and pesticides (panel d). The confusion matrices show the predicted exceedance on the x-axis against true exceedance on the y-axis of the test set, creating four quadrants: true negatives (top-left), false positives (top-right), false negatives (bottom-left) and true positives (bottom-right). The “0” label represents a record below the threshold and the “1” label represents a record that exceeds the threshold. From these confusion matrices, it can be seen that the majority of both the records above and below the exceedance thresholds are classified correctly. Due to the imbalance in the data, however, this also results in markedly more false positives than true positives in all four models.

The best-performing models all used probability thresholding as their data imbalance strategy. The selected probability thresholds, together with the sensitivity, specificity and accuracy are presented in Table 2 for each of the four contaminant groups. The probability thresholds reflect above which output probability the model classified the prediction as an exceedance. For each of the four contaminant groups, the probability thresholds were below 0.1, reflecting the highly imbalanced data. The selected probability thresholds were based on the highest average of sensitivity and specificity for the validation set, which resulted in a performance where the number of false negatives is reduced as much as possible while keeping the number of false positives in check. The sensitivity of the models ranged from 0.72 to 0.92, overall showing a good performance in the balance between true positives and false negatives. The specificity ranged from 0.59 to 0.81, with the dioxins model showing the lowest performance that deviated from the specificity and sensitivity scores of the other models. The dioxins model, however, did obtain the highest sensitivity score of 0.92. Relative to the models for the other three contaminant groups, the best threshold for the dioxins model prioritized a substantial reduction in false negatives, consequently resulting in a relatively larger number of false positives. Investigation of other thresholds on the validation set of the dioxins model showed that increasing the threshold sharply decreased the sensitivity, while only marginally increasing the specificity, leading to an overall smaller average. The specificity of the other contaminant groups showed good performance in the balance between true negatives and false positives with scores of 0.72, 0.8 and 0.81 for pesticides, mycotoxins and heavy metals, respectively. The accuracy of the models ranged from 0.61 to 0.81, showing a similar trend to the specificity. This similarity occurs as accuracy was mostly influenced by the true and false negatives, due to the imbalance in the data.

**Table 2:** The probability threshold, sensitivity, specificity and accuracy for the best performing model for each contaminant group on the test set (2023)

Contaminant group	Model performance			
	Probability threshold	Sensitivity	Specificity	Accuracy
Mycotoxins	0.01	0.72	0.80	0.80
Heavy metals	0.02	0.80	0.81	0.81
Dioxins	0.02	0.92	0.59	0.61
Pesticides	0.07	0.89	0.72	0.73

**Fig. 4:**
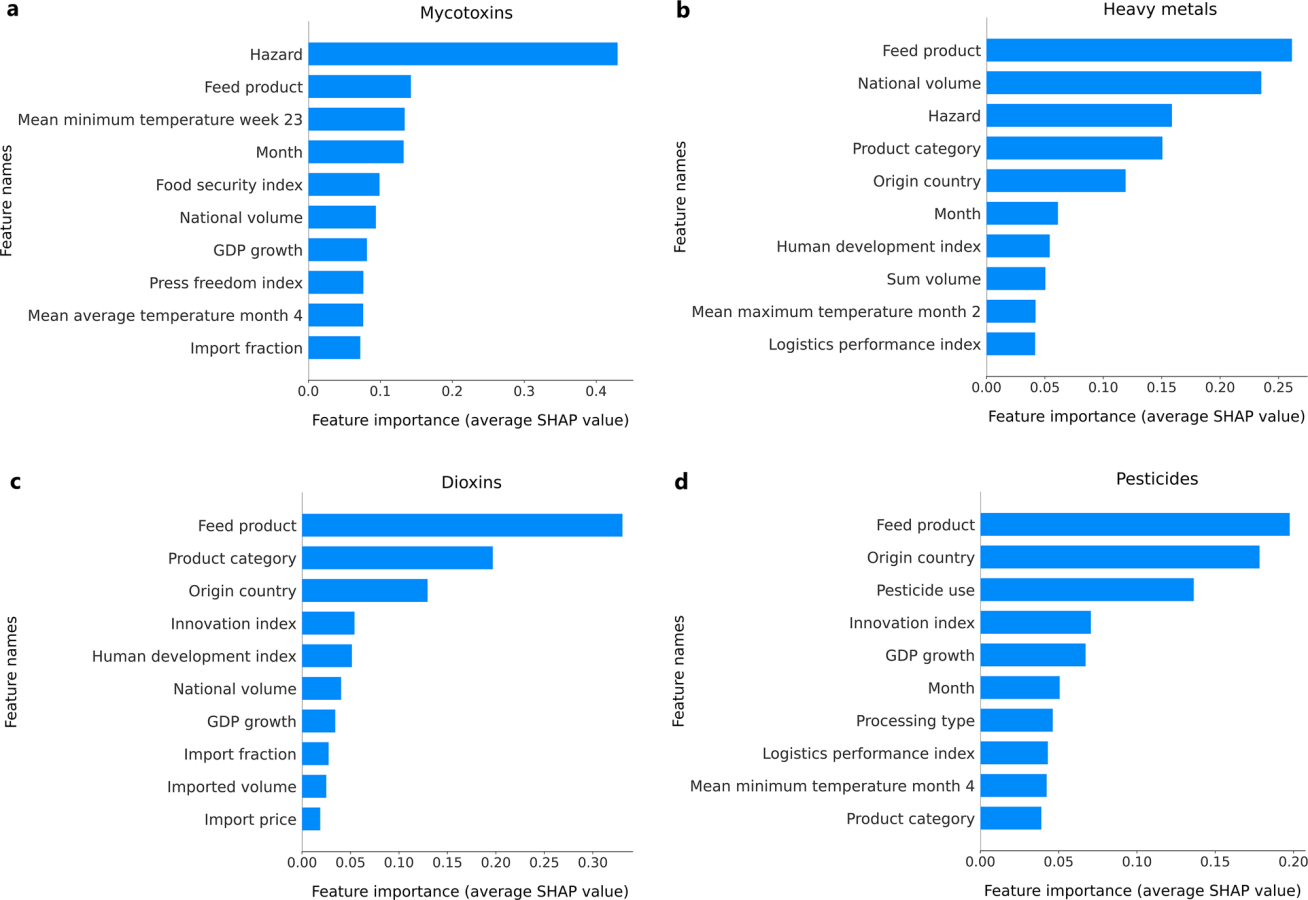
The top ten most important features determined with SHAP values. **a** The most important features for the mycotoxins model. **b** The most important features for the heavy metals model. **c** The most important features for the dioxins model. **d** The most important features for the pesticides model.

Figure 4 presents the top ten most important features for the prediction of the contaminant groups for mycotoxins (panel a), heavy metals (panel b), dioxins (panel c) and pesticides (panel d). For the mycotoxins model, the hazard (i.e., the specific mycotoxin) had the biggest influence on the prediction with a SHAP value of almost a factor three bigger than the second most important feature. The specific feed product, the mean minimum daily temperature in week 23 and the sampling month further influenced the prediction the most. For heavy metals, the feed product and national production volume had the most influence on the prediction, followed by the specific hazard (arsenic, lead, mercury or cadmium), product category and country of origin. In the case of dioxins, the feed product is the most important feature with quite a distance to the next features. Additionally, the product category and the country of origin have a big influence on the model prediction for dioxins. In the pesticides model, the feed product is once more the most important feature, together with the country of origin. Additionally, the pesticide use of the country of origin is an important feature, followed by the innovation index and the GDP growth of the country of origin.

### Decision support system

**Fig. 5:**
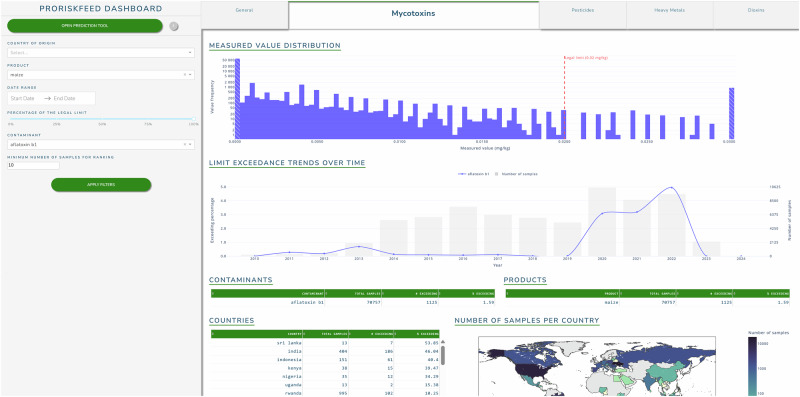
The data descriptive section of the dashboard. The tabs visualize a general overview of the data or the data of each of the contaminant groups, with optional filters on country of origin, feed product, data range, percentage of the legal limit, contaminant and minimum number of samples.

A DSS was developed to provide a user interface to experts for insights into the presence of contaminants in particular feed products sourced from specific countries of origin. The DSS was developed in the form of an interactive dashboard with two sections. The first section contained the descriptives of the monitoring data and is shown in Fig. 5. The visualizations provided information on measured concentrations, limit exceedances over time and number of records per contaminant and contaminant group, country and feed product. The user could view a general overview of the data across all contaminant groups or specifically visualize information on mycotoxins, pesticides, heavy metals or dioxins via the tabs on top of the dashboard. Filters could be set on the left side of the dashboard to investigate the data of a specific country, feed product, contaminant or date range. Furthermore, the user could select a minimum required number of records per selection and apply a custom percentage of the legal limit/guidance value for further inspection of the monitoring data. The figure shows an example selection of aflatoxin B1 in maize in the mycotoxins tab.

**Fig. 6:**
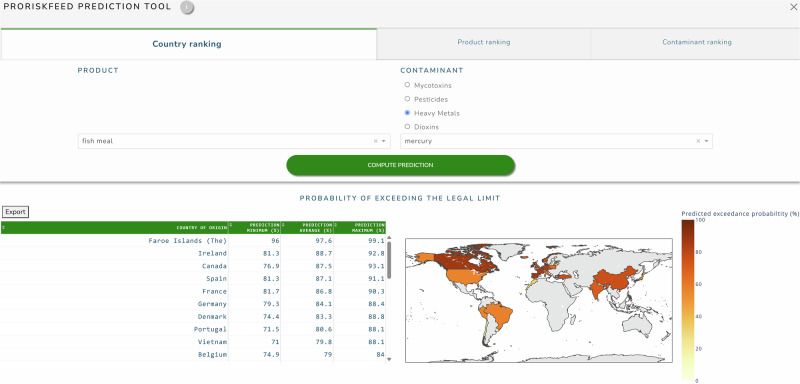
The predictions section of the dashboard. The tabs display the predicted probabilities of exceeding the predefined threshold for specific countries, feed products, or contaminants by making a selection in the other two options.

An example screenshot from the second section, which displayed the predictions from the machine learning models using the predefined exceedance thresholds, is shown in Fig. 6. This section was divided into three components: a country ranking, a product ranking and a contaminant ranking. A user could select a feed product and contaminant for the country ranking; a country and contaminant could be selected for the product ranking; and for the contaminant ranking, a feed product and country could be selected. After selection, a table was presented where the items were ranked from highest to lowest average probability of exceeding the predefined exceedance threshold. Besides the average probability, a minimum and maximum exceedance probability were also shown, reflecting the variation in probability over the different sampling months. The screenshot shows an example selection of mercury in fish meal in the country ranking component.

## Discussion

This study developed machine learning models with an average sensitivity of 83% and an average specificity of 73% to predict if a contaminant in a feed product from a specific country of origin exceeded a concentration threshold for mycotoxins, heavy metals, dioxins, and pesticides. Emphasis was placed on a holistic design, using a broad range of external drivers for emergence of food safety hazards as input into the machine learning models. In this way, both direct and indirect influences of these drivers on the emergence of the contaminants were considered. Such a holistic approach to the emergence of food safety hazards was originally proposed by the OECD^23^ and later applied by several others for food safety prediction models^17,21,24,25^. Our data-driven holistic prediction models can be used for decision making related to risk-based monitoring of food safety hazards in feed products, and routing and processing in the feed supply chain. The developed models are embedded in a DSS to create a user-friendly interface in which experts can quickly get an overview of the predictions, as well as the monitoring data used to develop the models.

Using machine learning, specifically the CatBoost algorithm, both the direct and indirect effects of the incorporated variables were considered, as well as their interrelationships. Model results showed that its predictions were mostly influenced by variables related to the contaminant, the feed products and the country of origin, but other variables were relevant as well, such as national and imported volumes of the feed product and characteristics of the country of origin. The latter variables showed the beneficial effects of the holistic approach to predict the emergence of the considered contaminants. A weather variable, namely the mean minimum daily temperature of week 23, was found to be one of the most important features in the mycotoxins model. This finding is consistent with previous studies showing that temperature is one of the main contributing factors to mycotoxin contamination^26–28^. This demonstrated that the models are able to capture the relationships between the input variables and hazard occurrence, which enables the prediction of trends related to a change in those variables, such as the impact of a changing climate on mycotoxins. The developed models showed to perform well and were validated on a year not seen during model training (i.e., the year 2023). By focusing the model performance on sensitivity and specificity, an emphasis was placed on reducing the number of false negatives as much as possible without creating too many false positives. As an end-user, one wants to miss as little true positives as possible. If accuracy would have been used as the guiding performance metric, the imbalance in the data would have caused the model to focus on reducing the number of false positives as there are much more negatives in the data. This would have resulted in a large number of false negatives as a consequence. Since correctly classifying the positive cases is important for risk-based monitoring, focusing on accuracy was therefore undesirable.

Due to the heavy imbalance in the data with the large majority of records being beneath the legal limits and guidance values, the models presented here were not developed using these limits or values as thresholds. This would have resulted in the models being biased towards predicting records below these thresholds to minimize their error during model training. This leads to poor performance in cases that are above the legal threshold, which for food safety are the most important to identify, and therefore creates models that would not be suitable in practice. By lowering the exceedance threshold to half of the legal limit or guidance value for mycotoxins, heavy metals and dioxins and by aggregating the different pesticides, we were able to create an acceptable trade-off between performance and usability. Additionally, although the sensitivity and specificity are dependent on the precise height of the threshold, other fractions than half of the limit were not considered to create a more straightforward threshold for our users. The current predictions can be used to identify records that have a higher probability of crossing the legal limits or guidance values as input for an improved risk-based monitoring that is data-driven, which can also benefit from this more conservative approach by including relevant early warning signals. Early warning signals will be missed if the legal limits or guidance values are used, while these signals are important to move from a reactive to a more proactive food safety monitoring.

In an earlier study, a semi-quantitative model named RiskFeed was developed to rank feed ingredients based on their estimated animal and human health risk, as related to the presence of certain chemical hazards in the feed ingredients^9^. To date, the RiskFeed model has been focusing on the chemical hazards of mycotoxins, dioxins, and heavy metals. It takes into account data related to five different variables: the presence of chemical hazards in feed ingredients, the use of feed ingredients for compound feed production, the inclusion rates in compound feed for various types of production animals, the country of origins of the feed ingredients, and the toxicity of the chemical hazards. The RiskFeed model focuses on ranking hazard-feed material combinations based on estimated risks to human health, whereas the presented models focus on predicting the presence of the hazards. Both provide valuable but different information to the end user. An advantage of the presented models is that they can provide predictions for the next year based on what was learned from the model variables from previous years.

Given the presented models were created with machine learning, updates using future monitoring data can be done relatively easily. With a further expansion of the database with more records, collected from all around the world, the models can be updated and will become more and more widely applicable. With further data collection, more emphasis should be given to the electronic recording of the country of origin, since more than a third of the current dataset contained records without the country of origin. With the adoption of the deforestation law in Europe mid-May 2023, recording of the country of origin becomes compulsory for a selected set of commodities^29^. Although feed materials are limited (to e.g., soy and palm oil) in this regulation, it is a start for more consistent recording of origin countries. In addition to missing data on the country of origin in the current dataset, the country of origin may not always have been recorded properly. This can be deduced from the fact that a relatively large share of the data were countries in Europe with a big harbor, such as the Netherlands, Germany, and Belgium. Feed materials are imported from non-European countries to Europe through these harbors, therefore the countries with the harbors were unlikely to be the original country of origin in all cases. For records related to very unlikely events, such as soy from the Netherlands (which more likely has been imported from South America to the Rotterdam harbor), the country of origin was changed to unknown. However, updating the country was not possible in all cases, because of plausible production possibilities. Since the set of holistic variables was linked to the country of origin, proper recording of origin country of the feed materials is very important, and may even further enhance the model performance.

Future work should also focus on trying to obtain more specific location information than just the country of origin, like the region of production or even coordinates of the farms and producing facilities. Having a more precise origin area enables more precise data for the used drivers, especially related to weather during production of the feed materials. A more specific location also gives the option of inclusion of other potential relevant drivers, like soil type, proximity to industry and possible pest exposures. More region- or farm-specific data, however, are hard to obtain and are often not collected via monitoring programs from companies and/or governments. To improve the performance of the prediction models, it is important that more of such data that are directly or indirectly related to the monitoring results will be collected in the future. Ideally, this would also include more details about the supply chain as a whole, so that the conditions around storage, transportation and processing can be taking into account as well.

To our knowledge, this study is the first to perform a prediction of the presence of multiple contaminants in a wide range of feed products on a global scale. It can be concluded that the use of machine learning with holistic drivers is successful for predicting the presence of contaminants in feed products. The presented models can be used by food safety authorities, feed producing companies and/or umbrella organizations for risk-based monitoring as well as for optimizing routing and processing in the feed chain. The embedding of the models and underlying historical monitoring data in the Decision Support System enables its user-friendly use and uptake in practice.

## Methods

### Monitoring data

Historical feed safety monitoring data were obtained for the period 2010–2023 from organizations in the animal feed industry in the Netherlands. Each record contains information related to the result of a chemical analysis of one sample of a feed material or animal feed (including pet feed ingredients), and comprises sampling date, analysis date, feed product, hazard, country of origin, limit of quantification (LOQ), exact concentration of the hazards including the corresponding unit, and the data provider. Since feed ingredients were imported to the Netherlands from around the globe, the data included analytical results from a wide variety of countries of origin. Results for four contaminant groups were included: mycotoxins, heavy metals, dioxins and pesticides, as far as regulated in European legislation.

### Socioeconomic data

Data on the socioeconomical status of all countries of origin present in the monitoring data were collected from various online data sources for the period 2010–2023. The socioeconomic variables included: the human development index, logistic performance index, corruption perceptions index, governance index, press freedom index, food security index, innovation index, the current gross domestic product (GDP) of a country and the GDP growth of a country (Table 3).

**Table 3:** Descriptions of the variables used in the machine learning models with their data sources

Model variables	Description	Data sources
Month	Month of sampling (or month of analysis, if sampling date is unavailable)	Historical monitoring data
Feed product	Product name	Historical monitoring data
Contaminant group	Contaminant group name (mycotoxins, heavy metals, dioxins or pesticides)	Historical monitoring data
Contaminant	Contaminant name	Historical monitoring data
Origin country	Country of origin	Historical monitoring data
Origin continent	Continent of origin	Inferred from country of origin
Product category	Category of the feed product according to EU No 68/2013	Based on the feed product name
Processing type	Processing type of a product (raw commodity, primary processing, secondary processing)	Based on the feed product name
Imported volume	Imported volume (tons) of the product in the sampling year from the country of origin by the Netherlands	Eurostat
National volume	National production volume (tons) of the product in the Netherlands in the sampling year	CBS Statline and branch organizations
Sum volume	Sum of imported volume from the country of origin and the national volume in the sampling year	Based on imported and national volume
Import fraction	Imported volume from the country of origin divided by the sum volume in the sampling year	Based on imported and national volume
Price per ton (import)	Price (euro) of the imported volume divided by the imported volume from the country of origin in the sampling year	Eurostat
Pesticide use	Agricultural use of pesticides (tons) for the country of origin in the sampling year	FAOstat
Corruption perceptions	The Corruption Perceptions Index (CPI) for the country of origin in the sampling year	Transparency International
Democracy	The Democracy Index for the country of origin in the sampling year	Economist Intelligence
Human development	The Human Development Index for the country of origin in the sampling year	United Nations
Logistics performance	The Logistics Performance Index for the country of origin in the sampling year	World Bank
Governance	The Governance Index for the country of origin in the sampling year	World Bank
Press freedom	The Press Freedom Index for the country of origin in the sampling year	Reporters Without Borders (RSF)
GDP current	The Gross Domestic Product (U.S. dollar) for the country of origin in the sampling year	World Bank
GDP growth	The economic growth (%) for the country of origin in the sampling year	World Bank
Food security	The Global Food Security Index for the country of origin in the sampling year	Economist Impact
Innovation	The Innovation Index for the country of origin in the sampling year	World Intellectual Property Organization
Max temperature	Mean of the daily maximum temperature (°C) for the country of origin for the 6 months before the harvest (weekly/monthly)	Copernicus
Mean temperature	Mean of the daily mean temperature (°C) for the country of origin for the 6 months before the harvest (weekly/monthly)	Copernicus
Min temperature	Mean of the minimum daily temperature (°C) for the country of origin for the 6 months before the harvest (weekly/monthly)	Copernicus
Mean precipitation	Mean of the daily precipitation (mm) for the country of origin for the 6 months before the harvest (weekly/monthly)	Copernicus
Relative humidity	Mean of the daily relative humidity (%) for the country of origin for the 6 months before the harvest (weekly/monthly)	Copernicus
Exceedance of the threshold (output variable)	A binary variable (0/1) representing whether the measurement exceeded the predefined threshold (based on the legal limit or guidance level)	Based on historical monitoring data and legislation

Furthermore, data on Dutch feed production, trade volumes and trade prices were collected. Information about the production of feed materials in the Netherlands was obtained from CBS Statline (https://opendata.cbs.nl/statline/#/CBS/nl/dataset/85636NED) and through communication with Dutch branch organizations, e.g., the Netherlands Oils and Fats Industry. Data on feed trade volumes and feed trade prices were collected from Eurostat, the statistical office of the European Union, on a country level. Data were selected from the “EU trade since 1988 by HS2-4-6 and CN8 (DS-045409)” dataset (https://ec.europa.eu/eurostat/comext/newxtweb). Trade volumes (in tons) and corresponding prices (in euros) were collected from all countries (including individual countries in the EU) that exported animal feed to the Netherlands in the period of the retrieved monitoring data (2010-2023). The Netherlands was selected as the reporter country and all other available countries were selected as partner countries. An overview of the Combined Nomenclature (CN) codes used for the feed product selection can be found in the Supplementary information (Table S1)^30^. Not all products in the monitoring data could be linked to a CN code, only records with a CN code were extended with trade data.

### Weather data

To link weather data with the correct year of cultivation of the feed material, the harvest periods were collected per crop and country of origin combination. These harvest periods were retrieved from AGRI4CAST from Joint Research Centre of the European Commission, Center for Sustainability and the Global Environment (SAGE), Foreign Agricultural Service from U.S. Department of Agriculture (USDA), and Food and Agriculture Organization of the United Nations (FAO). Harvest dates were only retrieved for raw commodities and not for processed feed due to their multiple ingredients and longer shelf lives.

Weather data were retrieved from the Copernicus Climate Change Service^31^. For all countries of origin, the minimum, mean and maximum daily temperatures of all days from the assumed harvest dates of the feed materials to 6 months prior this date were collected. In addition, the daily precipitation and relative humidity of all the days from the assumed harvest dates to 6 months prior these dates were collected. To avoid adding a disproportionate amount of weather features, which could lead to overfitting, a balance between their number and granularity needed to be reached. It was therefore decided to aggregate the weather data. For the first 4 months of the collected period, the different temperatures, precipitation and relative humidity per country of origin were averaged per month. For the 2 months closest to the assumed harvest date, these data were averaged per week.

### Data processing

All records from the retrieved historical monitoring data were combined in one dataset, which was saved and processed in a PostgreSQL database. Duplicates (i.e., monitoring data records provided by multiple organizations) were removed based on sample number, date of sampling, product, contaminant and analysis result (converted to mg/kg), after which the data were anonymized. The feed products were harmonized and classified in line with the Catalog of feed materials (EU No 68/2013). For records with a combination of a feed product and a country of origin that did not occur in the FAO agricultural production dataset (https://www.fao.org/faostat/en/#data), the country of origin was set to unknown to avoid learning from incorrect data. Additionally, a processing type was added for each product. The processing type was set to “raw commodity” (e.g., maize), “primary processing” for minimally processing such as milling and drying (e.g., maize flour), or ‘secondary processing’ for more advanced processing (e.g., maize gluten feed)^1^.

Each record in the monitoring dataset was supplemented with maximum limits or guidance values defined in European legislation. The maximum limits for the presence of the considered contaminants in feed were derived from Directive 2002/32/EC. The guidance values for the presence of other mycotoxins than aflatoxin B1 in feed were derived from Commission Recommendations 2006/576/EC and 2013/165/EU. The maximum residue limits (MRLs) for pesticides were obtained from the EU pesticides database (https://ec.europa.eu/food/plant/pesticides/eu-pesticides-database/start/screen/mrls). In all cases, the applicable legislation in the year 2023 was used; changes in legislation over time (in the period considered, from 2010-2023) were not taken into account to obtain a single legal limit or guidance value to compare the analytical results to across the years. This was done to keep the pattern of exceedance in the data consistent. Records with a LOQ higher than the maximum limit or guidance value were removed from the dataset.

The socioeconomic variables were linked to the historical monitoring data based on country of origin, product and sampling year. Missing values in the socioeconomic variables, due to values for some years not being available for some indices, were imputed with linear interpolation of the values of the surrounding years. Additionally, the values of the socioeconomical variables were categorized into four different categories (“low”, “medium”, “high”, “very high”). The categories were manually created by dividing the data into intervals containing roughly the same number of data points while keeping a logical spread across the values of the variable. This categorization allowed for easier processing by the model, increasing performance. The weather data were linked to the monitoring records based on commodity name and sampling date, where the weather of the harvest prior to the moment of sampling was selected.

The descriptions of all variables in the dataset, together with their data sources, can be found in Table 3.

### Contaminant thresholds

A major part of the historical monitoring data referred to analytical results with concentrations below the European legal limits and guidance levels for the contaminants: 98.8% for mycotoxins, 99.6% for heavy metals and dioxins, and 99.9% for pesticides. This imbalance in data is difficult to handle in machine learning model development, since there is insufficient information in the data to uncover the patterns for cases above the legal limit/guidance value. Therefore, a lower threshold for contaminant presence was chosen when developing the machine learning models. For mycotoxins, heavy metals and dioxins, half of the respective legal limit or guidance levels of the contaminants was considered as the predefined threshold. Additionally, it was decided to not include dioxin-like polychlorinated biphenyls in the dioxins group, since the inclusion of these chemicals lowered the percentage of records above the threshold by more than half. For pesticides, the analytical results for all pesticides analyzed in one sample were aggregated due to the large imbalance and the substantial number of measured pesticides. If one or more of the pesticide residue levels in a sample were found to be above the respective MRL, this sample was considered as exceeding the threshold. This approach for pesticides was defined in consultation with experts: Since pesticides are not a naturally occurring hazard as they are purposely added by humans and are analyzed using a multimethod for their identification, it is often sufficient for risk-based monitoring to know if any pesticide residue level will occur above an MRL.

A model output variable representing the exceedance of the contaminant threshold (yes/no) was added to the dataset, reflecting the presence of that contaminant. This was done by comparing the observed concentration of the contaminant (analytical result) with the above mentioned threshold for each record.

### Machine learning models

Machine learning models were developed to predict whether the contaminants in feed from a specific country of origin exceeded their respective predefined threshold. Models were built separately for each of the four contaminant groups: dioxins, pesticides, mycotoxins and heavy metals. Model input consisted of the variables from Table 3. The model output consisted of the probability that a sample from a batch of feed exceeded the predefined threshold, which was transformed into a 0 (under the threshold) or 1 (above the threshold) based on the value of the predicted probability.

The dataset was split into three different sets for model development: a training set, a validation set and a test set. The training set consisted of the data ranging from 2010–2021. The validation set and test set consisted of data from 2022 and 2023, respectively, to assess the model’s generalizability by evaluating the performance on years not used for model training. The model performance was evaluated using confusion matrices, sensitivity and specificity. A confusion matrix consists of the number of True Negatives (TN), False Positives (FP), False Negatives (FN) and True Positives (TP). Sensitivity (also referred to as recall) is the fraction of positive cases that are correctly predicted as positive, while specificity is the fraction of negative cases that are correctly predicted as negative. Sensitivity and specificity are subsequently defined as follows:

1 $${Sensitivity}=\frac{{TP}}{{TP}+{FN}\,}$$

2 $${Specificity}=\,\frac{{TN}}{{TN}+{FP}}$$

Multiple algorithms were initially applied to the data, including logistic regression, support vector machine, naive Bayes, Bayesian network, random forest, gradient boosting, XGBoost, CatBoost, and TabNet. From these algorithms, CatBoost showed to perform best and was chosen as the algorithm for the final model development. CatBoost is an algorithm that creates an ensemble of decision trees and has been specifically designed to handle categorical data^32^. The binary splits in the decision trees are chosen such that the error in the prediction of a tree is minimized. The trees in CatBoost are sequentially generated, where each tree tries to compensate for the errors made by the previous tree. The final prediction is created by averaging the output of each decision tree from the ensemble. An advantage of CatBoost is that it is able to handle missing data automatically. This is relevant as there are missing values for several variables in our data, such as the country of origin and the trade volumes. CatBoost treats the missing data as a separate category per variable when creating the splits in the decision tree, which means it can learn based on the error and the relationships in the data how to best incorporate them in the model.

Five-fold cross-validation was used on the training set to determine the best hyperparameter values for the models. Each fold contained a distinct set of years to avoid leaking trends from those years to other folds. The years were distributed as follows: 2010–2011–2012, 2013–2014–2015, 2016–2017, 2018–2019 and 2020–2021. Hyperparameters that were varied in model training were learning rate (tested values: 0.01, 0.05, 0.1), maximum tree depth (tested values: 6, 8, 10), and L2 regularization (tested values: 10, 25, 50, 75, 100). The learning rate influences how much each individual decision tree affects the final prediction, the maximum tree depth controls how many splits the decision trees can maximally have, and the L2 regularization reduces overfitting. The tested values for these hyperparameters were selected based on recommendations from the CatBoost documentation (https://catboost.ai/docs/en/concepts/parameter-tuning) and manual exploration. The best combination of hyperparameters was selected based on the highest area under the Receiver Operating Characteristic (ROC) curve, which is a curve of the sensitivity against 1-specificity. Strategies to deal with the imbalanced nature of the data were tested, and included oversampling, undersampling, class weighting and probability thresholding. Probability thresholds were chosen by selecting the threshold with the highest average of the sensitivity and specificity to find a good balance between the percentage of false negatives and false positives. The results from the different imbalance strategies were evaluated and selected based on their performance on the validation set. A final model was trained on the combined data from 2010–2022 with the best hyperparameters and imbalance strategy and applied on the test set (2023) for each contaminant group. The feature importance of the different variables in the models were determined to assess the impact of each of the variables on the model predictions using SHAP values. SHAP values represents the average marginal contribution of a variable on the model prediction over all possible combinations of variables^33^.

All code was created in the programming language Python (version 3.9.10).

### Decision support system development

A DSS was developed to provide a user interface to experts and present an easy-to-understand overview of the data and the predictions from the machine learning models. The DSS included two sections. The first section showed data descriptives of the monitoring data with visualizations that can be tailor-made, based on selections from the end user. The second section provided the predicted probabilities that the concentration of a contaminant in a specific feed product from a specific country exceeded the predefined threshold value.

The DSS was created in consultation with experts from the animal feed industry to allow them to shape the visualizations, filter options and prediction output to make the DSS useful in their daily operations. It was applied in their organization in the year 2024 to test its use in practice and provide feedback on the interface design.

The DSS was built in Python (version 3.9.10) and connected directly to the data stored in the PostgreSQL database.

## Supplementary information

Supplementary Information

## Data Availability

The monitoring data presented in the study are confidential data from the animal feed industry and therefore not available.

## Code availability

The code for the machine learning models is available upon request from the corresponding author.
