# Supplementary material for: Early warning predictions of contaminants in animal feed using machine learning
Source: NPJ Sci Food. 2025 Nov 21;9:279. doi: 10.1038/s41538-025-00634-1 (PMC12749966; doi:10.1038/s41538-025-00634-1)
Supplement: Supplementary file 1 — Supplementary Information [file 41538_2025_634_MOESM1_ESM.pdf]

Supplementary information

Table S1: The available Combined Nomenclature codes used per product category and subcategory.

Product category	Product subcategory	CN8 codes*
Cereal grains and products derived thereof	wheat	10019900
	barley	10039000
	maize	10059000
	triticale	10086000
	rye	10029000
	sorghum	10079000
	oats	10049000
	buckwheat/millet	10081000, 10082900
	wheat products	23023010, 23023090
	maize products	23021010, 23021090
	rice products	23024002, 23024008
	products other cereal grains	23024010, 23024090
	ddgs	23033000
	maize germ meal/expeller	23069005
	maize gluten feed	23031019
	maize gluten meal	23031011
Oil seeds, oil fruits, and products derived thereof	soya bean meal/expeller	23040000
	sunflower seed meal/expeller	23063000
	coconut/copra meal/expeller	23065000
	palm kernel meal/expeller	23066000
	rapeseed meal/expeller	23064100, 23064900
	linseed meal/expeller	23062000
	groundnut meal/expeller	23050000
	cottonseed meal/expeller	23061000
	soya hulls	23080090
	soya bean	12019000, 12081000
	rapeseed	12051090, 12059000
	linseed	12040090
	sunflower seed	12060091, 12060099
	miscellaneous seeds	12079996
	coconut fat	15131110, 15131930
	palm oil	15111010, 15119091
	palm kernel fat	15132110, 15132930
	other plant oils	15141110, 15141910, 15121110, 15121910, 15151100, 15151910, 15180039, 15180099
	soya bean oil	15071010, 15079010
Legume seeds and products derived thereof	pea (products)	07131090
	lupines	12092950

	beans	07133100, 07133200, 07133390, 07135000
Other plants, algae and products derived thereof	algae	12122100, 12122900
Tubers, roots, and products derived thereof	manioc	07141000
	dried beet pulp	23032010, 23099091
	sugar beet molasses	17039000
Forages and roughage, and products derived thereof	grass-/clover-/alfalfa meal	12141000
Other seeds and fruits, and products derived thereof	citrus pulp	23080040
	sugar cane molasses	17031000
Products and by-products obtained by fermentation using micro-organisms, inactivated resulting in absence of live micro-organisms	vinasses	23032090
Milk products and products derived thereof	whey powder/wpc	04041002, 04041004, 04041006, 04041012, 04041014, 04041016
	milk powder/-concentrate	04021019, 04021099
Land animal products and products derived thereof	animal proteins	05059000, 23011000
	other animal fats	15021010, 15029010, 15011010, 15012010, 15060000
	poultry fat	15019000
Fish, other aquatic animals and products derived thereof	fish meal	23012000
	fish oil	15042090
Minerals and products derived thereof	lime/limestone	28365000
	salt	25010091, 25010099
	sodium-bicarbonate	28363000
	calcium phosphate (incl. org.)	28352200, 28352500, 28352600
	magnesium oxide	25199010
	l-lysine hcl	29224100
	dl-methionine	29304090
	copper salts	28332500, 28255000
	clay minerals	25070020, 25070080, 25081000
Miscellaneous	organic acid	29151100, 29154000, 29155000, 29163100, 29181100, 29181400
	enzymes	35079090

* CN8-codes obtained from Regulation (EEC) 2658/87[1]

[1] https://eur-lex.europa.eu/eli/reg/1987/2658
